# The analysis of *Lactobacillus* spp. distribution in the vaginal microbiota of Polish women with abnormal Pap smear result

**DOI:** 10.3389/fmicb.2023.1257587

**Published:** 2023-11-09

**Authors:** Karolina Frąszczak, Bartłomiej Barczyński, Radosław Siwiec, Adrianna Kondracka, Anna Malm, Jan Kotarski, Elzbieta Witt, Izabela Korona-Głowniak

**Affiliations:** ^1^I^st^ Department of Oncological Gynaecology and Gynaecology, Medical University in Lublin, Lublin, Poland; ^2^Department of Pharmaceutical Microbiology, Faculty of Pharmacy, Medical University in Lublin, Lublin, Poland; ^3^Department of Obstetrics and Pathology of Pregnancy, Medical University in Lublin, Lublin, Poland; ^4^Independent Laboratory of Cancer Diagnostics and Immunology, Medical University in Lublin, Lublin, Poland; ^5^Frauenklinik, Marienhospital Witten, Witten, Germany

**Keywords:** vaginal microbiota, *Lactobacillus*, *L. gasseri*, *L. crispatus*, HPV, cervical cancer

## Abstract

**Introduction:**

A healthy vaginal microbiota is represented mainly by *Lactobacillus* spp. and plays a vital role in maintaining the functional balance in the vaginal environment. Scientists have drawn attention to possible correlations between the vaginal microbiome and gynecological neoplasms. Several recent studies have shown a potential link between the vaginal microbiome and the risk of developing cervical cancer from human papillomavirus (HPV) infection. This study aimed to compare the prevalence and abundance of various lactic acid bacteria species (LABs) in vaginal swabs from healthy controls and patients with abnormal Pap smear results.

**Methods:**

The study included 100 women (79 patients with abnormal cervical Pap smear results and 21 controls) from whom vaginal swabs were collected. Real-time quantitative PCR was used to determine seven lactic acid bacteria (LAB) species and their quantities.

**Results:**

Most patients were colonized by two *Lactobacillus* species, primarily *Lactobacillus gasseri* (93%) and *L. crispatus* (83%). Patient age and place of residence were associated with the diversity of LAB in the vaginal microbiota. The abundance of *L. delbrueckii* in the vaginal microbiota increased, whereas the abundance of *L. gasseri* abundance decreased, with patient age. *Lactobacillus acidophilus* and *Limosilactobacillus fermentum* were significantly more often detected in patients living in rural versus urban areas. Statistical analysis did not show any significant differences in LAB between groups of patients with various changes on smear tests.

**Discussion:**

The degree of dysplastic changes in the endothelium or the presence of a group of atypical cervical stratified epithelial cells was not associated with significant changes in the studied vaginal bacteria.

## 1. Introduction

Bacteria, viruses, archaea, yeast, and fungi form the human microbiome (Marchesi and Ravel, 2015; Sender et al., 2016; Requena and Velasco, 2021), which colonizes the skin, genitourinary system, oral cavity, and intestinal tract. The human microbiota establishes a symbiotic relationship with the host and plays a vital role in maintaining the host’s physiological homeostasis (Dominguez-Bello et al., 2019). The composition of the microbiota differs depending on the site of microbial colonization. A healthy vaginal microbiota is represented mainly by *Lactobacillus* spp. including *L. crispatus*, *L. jensenii, L. gasseri,* and *L. iners* (Requena and Velasco, 2021), whereas *Prevotella*, *Gardnerella*, *Atopobium*, *Sneathia*, *Bifidobacterium*, *Megasphaera*, and *Anaerococcus* are associated with vaginosis (Moreno and Simon, 2018). The microbiota plays a vital role in maintaining functional balance in the vaginal environment and in preventing colonization by pathogens and the overgrowth of commensal microorganisms. *Lactobacillus* spp. stabilize the vaginal microbiota by producing antimicrobial components such as hydrogen peroxide, lactic acid, and bacteriocin-like compounds. The ability to adhere and compete with other harmful microorganisms is also crucial (Borges et al., 2014; Tachedjian et al., 2017). In most areas of the human body, a highly species-diverse microbiome is a sign of health (Turnbaugh et al., 2007; Flores et al., 2014). However, for the female reproductive system, a healthy state is more often associated with low microbial diversity and the dominance of one or more *Lactobacillus* species (Ravel et al., 2011; Liu et al., 2013; MacIntyre et al., 2015). An increasing number of publications indicate a correlation between disturbances of the vaginal microbiota, bacterial vaginosis, and the development of fungal, bacterial, and viral infections (Marchesi and Ravel, 2015; Mitra et al., 2016; Sender et al., 2016; Requena and Velasco, 2021). Some studies have demonstrated a correlation between bacterial vaginosis and increased persistence of human papillomavirus (HPV) infections as well as the development of cervical intraepithelial neoplasia (Gillet et al., 2011; King et al., 2011; Gillet et al., 2012; Guo et al., 2012; Champer et al., 2018). Women with persistent HPV infections have a more species-diverse microbiome, rich in *L. gasseri* and *Gardnerella vaginalis* and at the same time with a low abundance of *Lactobacilli* (Gao et al., 2013; Lee et al., 2013). The rate of resolution of HPV infection and development cervical cancer might also depend on the composition of the vaginal microbiome (Brotman et al., 2014). Apart from vaginal microbiota, also cervical microbiome is important for female’s health. Higher cervical species diversity has been demonstrated in severer cervical intraepithelial neoplasia of reproductive-age women (Wu et al., 2021). More complex composition of cervical microbiota which tends to progress with the aggravation of lesions as well as the prevalence of anaerobic bacteria were found in women with cervical pathology. Disturbed cervical microbial community is associated with cervical intraepithelial neoplasia and cervical cancer (Wang et al., 2022). Lower relative abundance of *Lactobacillus* as well as presence of *Porphyromonas*, *Prevotella* and *Campylobacter* and *Sneathia* have been suggested to promote the development of cervical pathology.

Cervical cancer is the fourth most common cancer in the world in terms of both incidence and mortality. In 2018, nearly 570,000 new patients were diagnosed globally, and 311,000 women died from cervical cancer (Arbyn et al., 2020). This cancer can be prevented through early diagnosis and treatment of precancerous states, but it remains the most common cancer in most low and middle-income countries (Arbyn et al., 2020). This study aimed to compare the distribution of 7 various vaginal *Lactobacillus* spp. between Polish women with abnormal Pap smear results (study group) and healthy individuals (control group) based on quantitative analysis of bacteria in vaginal swabs. We also assessed correlations between the vaginal microbiota and patient age, place of residence (urban vs. rural), HPV status, menopausal status, and history of vaginal infection within the past 3 months.

## 2. Materials and methods

### 2.1. Participants

The study included 100 patients (aged 18–72 years) from Ist Department of Gynecological Oncology and Gynecology, Medical University in Lublin, Poland. Inclusion criteria were admission to Gynecology Department and expressed willingness to participate in the project. Exclusion criteria were lack of consent, undergoing surgical procedures in the lower genital area, age below 18, inability to collect accurate data for the project. Patients with abnormal Pap smear results were included in the study group, and healthy individuals were used as a control group. TBS system, which provides a uniform format and standardized lexicon for cervical/vaginal cytology reports was used to classify abnormal Pap smears (Chatterjee et al., 2000). Vaginal swabs were collected from all study participants from October 2014 to November 2017. Considering the size of women population aged 18–72 years in Poland is approximately 9.5 mLn, the margin of error of the measured value at the 95% confidence interval is ±6% when tested 100 women.

This study was approved by the Bioethics Committee of Medical University in Lublin KE – 0254/174/2014 and was conducted in accordance with the fundamental ethical principles described in the Declaration of Helsinki (World Medical Association, 2013). All participants signed informed consent to participate in this study.

### 2.2. Vaginal swabs for genetic testing

Vaginal swabs were collected from patients using sterile silicone brushes (Genomic Micro AX Swab Gravity Plus kit, A&A Biotechnology). Swabs were placed in 1.5 ml tubes with 700 μL Spheroid Lysis Buffer (SLB) (A&A Biotechnology). All specimens were stored in ultralow freezer at -80°C until further analyses. DNA isolation was performed within 6 months from the date of swab collection. The extraction resulted in high-quality DNA that is free of inhibitors for reproducible results.

### 2.3. Real-time quantitative PCR

Real-time quantitative PCR was performed on 100 samples of DNA isolated from vaginal swabs from 79 patients with abnormalities in the smear test (50 patients with low-grade and 29 patients with high-grade epithelial lesions) and 21 healthy patients (control group) using LightCycler 96 (Roche). The quantitative analysis included seven *Lactobacillus* species (*Lactobacillus acidophilus*, *Lactobacillus crispatus*, *Lactobacillus delbrueckii*, *Limosilactobacillus fermentum*, *Lactobacillus gasseri*, *Lactiplantibacillus plantarum*, and *Lacticaseibacillus rhamnosus*). The quality control (QC) of the implementation procedure included IC (internal control) with amplification for the IC within normal limits, HC (high copy) control, LC (low copy) control, a normal control (NC) with amplification for IPC, and NTC (no template control) with no amplification. Standards, positive controls, and negative controls were performed in triplicate for each analysis. The primers (Genomed, Warsaw, Poland) presented in Table 1 were used for the detection and quantification of the seven LAB species.

**Table 1:** Sequences of primers used in real-time PCR reactions.

	Primer F	Primer R	Reference
*L. acidophilus*	GGRTGATTTGTTGGACGCTAG	GCCGCCTTTCAAACTTGAATC	Haarman and Knol (2006)
*L. crispatus*	TGGAAACAGRTGCTAATACCG	CAGTTACTACCTCTATCTTTCTTCACTAC	Byun et al. (2004)
*L. delbrueckii*	TGGATCACCTCCTTTCTAAGGAAT	TGTTCTCGGTTTCATTATGAAAAAATA	Zhang et al. (2012)
*L. fermentum*	TGCTTGCATCTTGATTTAATTTTG	GGTTCTTGGATYTATGCGGTATTAG	Zhang et al. (2012)
*L. gasseri*	GAAAGAGCCCAAACCAAGTGATT	CTTCCCAGATAATTCAACTATCGCTTA	Byun et al. (2004)
*L. plantarum*	AGCGAGCGGAACTAACAGATTTAC	AGCTGATCATGCGATCTGCTT	Haarman and Knol (2006)
*L. rhamnosus*	GCACCTGATTGATTTTGGTCG	GTCCATTGTGGAAGATTCCC	Zhang et al. (2012)

### 2.4. Statistical analysis

The Shapiro–Wilk test was used to check the normality of the data distribution. The verification of statistical hypotheses was based on non-parametric tests. The non-parametric Mann–Whitney test was used to compare the two groups (study and control). The non-parametric Kruskal-Wallis test was used to compare more than two groups (if this test showed a statistically significant difference between the groups, the post-hoc test was used for further comparisons). Spearman’s rank correlation coefficients were calculated to analyze the coexistence of various bacterial species. The Pearson Chi-square test and Fisher’s exact test were used to evaluate the relationship between two qualitative features. STATISTICA 13.1 was used to perform statistical analyses. A *p* value of <0.05 was considered statistically significant.

## 3. Results

### 3.1. Clinical background of the study subjects

**Figure 1:**
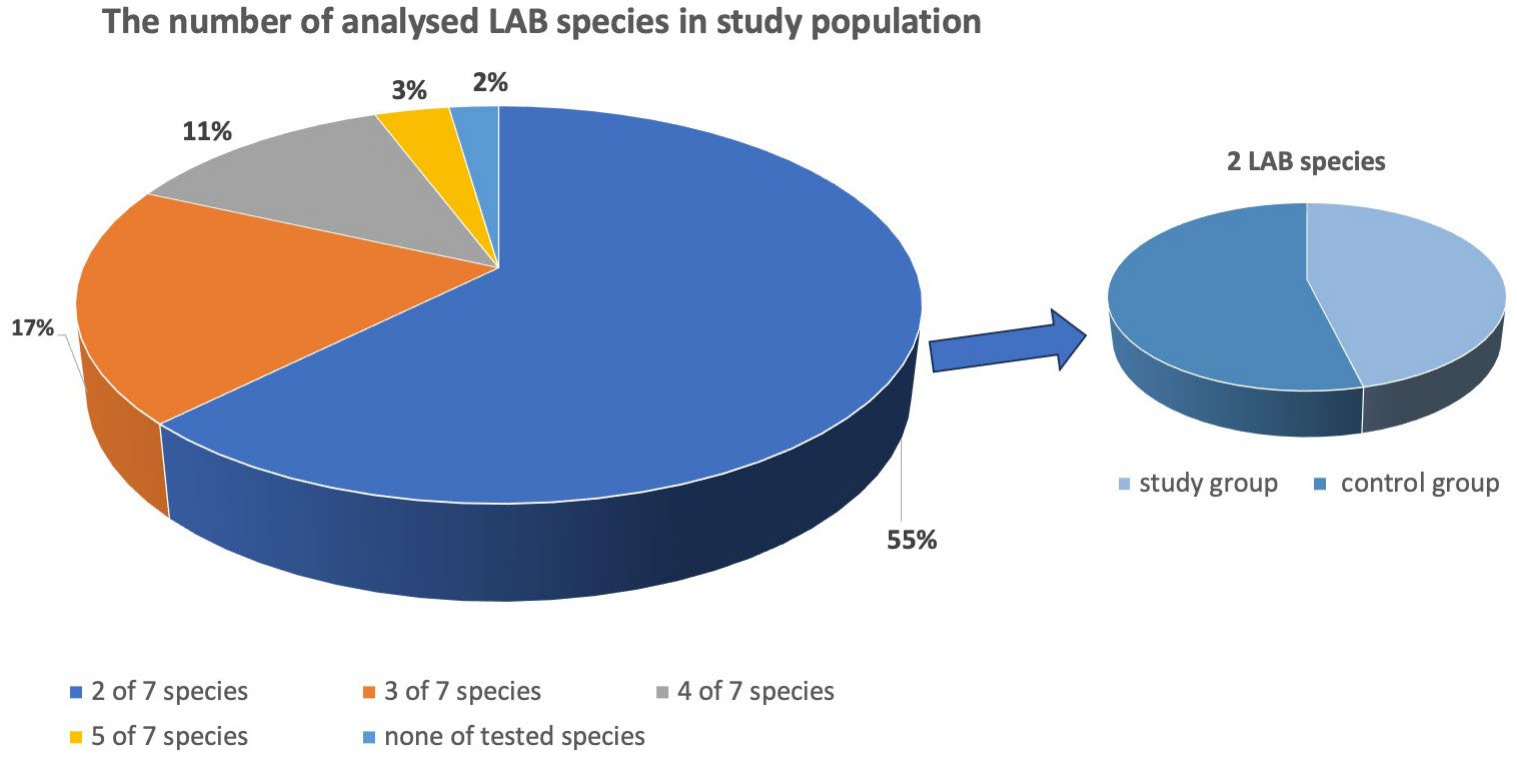
Number of analyzed LAB species in study participants.

In our study, most females were premenopausal. Over 50% of study participants have not been tested for HPV infection and none of them have been vaccinated against it. Vulvovaginitis within lat 3 months was reported in 28% of enrolled females. The results of smear test were normal in 21% of participants, ASC-US was found in 24%, ASC-H in 11%, LSIL in 26% and HSIL in 18% of females. The demographic and clinical data of participants are summarized in Table 2. The vaginal microbiota of most participants (55%) was dominated by two of the seven LABs investigated. Three species were observed in 17% of the examined participants, four in 11%, and five in 3% of participants. In 2% of patients, none of the studied LABs was found. Two LABs tested were detected in 53.16% of the vaginal swabs collected from patients with abnormal Pap smear results (study group) and in 61.91% of individuals from the control group (Figure 1).

**Table 2:** Demographic and clinical data of the enrolled participants.

Parameters	*N* (%)
Place of residence	
City	33
Village	67
Menopausal status	
Postmenopausal	18
Premenopausal	82
TBS diagnosis	
Normal	21
Atypical squamous cells of undetermined significance	24
Atypical squamous cells, cannot exclude a high-grade squamous intraepithelial lesion	11
Low-grade squamous intraepithelial lesion	26
High-grade squamous intraepithelial lesion	18
Human papillomavirus diagnosis	
Positive	23
Negative	23
Unknown	54
Vulvovaginitis within the last 3 months	
Occurred	28
Not occurred	72
Vaccination against human papillomavirus	
Yes	0
No	100

TBS, The Bethesda System for Reporting Cervical Cytology.

### 3.2. Associations between clinical findings and *Lactobacilli*

There were no statistically significant differences in identified LAB species between groups of patients with normal Pap smear results and those with ASC-US, ASC-H, LSIL, and HSIL (*p* > 0.05). In general, *L. gasseri* and *L. crispatus* were the most common LAB species detected in all enrolled patients (Table 3). *L. gasseri* and *L. crispatus* were also most detected in patients with ASC-US, ASC-H, LSIL, and HSIL. The occurrence of LAB species in participants with various Pap smear results is summarized in Table 3.

**Table 3:** Prevalence of LAB species detected in enrolled participants in relation to cervical Pap smear result.

	Total *n* = 100	Negative Pap smear (normal) *N* = 21	LSIL *N* = 26	HSIL *N* = 18	ASC-US *N* = 24	ASC-H *N* = 11	*p* value
*N* (%)							
*L. acidophilus*	2 (2.0)	4 (4.8)	0 (0.0)	1 (5.6)	0 (0.0)	0 (0.0)	0.52
*L. crispatus*	83 (83.0)	17 (81.0)	23 (88.5)	16 (88.9)	18 (75.0)	9 (81.2)	0.71
*L. delbrueckii*	25 (25.0)	6 (28.6)	7 (26.9)	6 (33.3)	4 (16.7)	2 (18.2)	0.72
*L. fermentum*	9 (9.0)	2 (9.5)	3 (11.5)	2 (11.1)	2 (8.3)	0 (0.0)	0.67
*L. gasseri*	93 (93.0)	20 (95.2)	24 (92.3)	16 (88.9)	16 (91.7)	11 (100)	0.69
*L. plantarum*	4 (4.0)	1 (4.8)	2 (7.7)	0 (0.0)	1 (4.2)	0 (0.0)	0.54
*L. rhamnosus*	15 (15.0)	5 (23.8)	4 (15.4)	1 (5.6)	4 (16.7)	1 (9.1)	0.54

LSIL, low-grade squamous intraepithelial lesion; HSIL, high-grade squamous intraepithelial lesion; ASC-US, atypical squamous cells of undetermined significance; ASC-H, atypical squamous cells, cannot exclude a high-grade squamous intraepithelial lesion.

**Figure 2:**
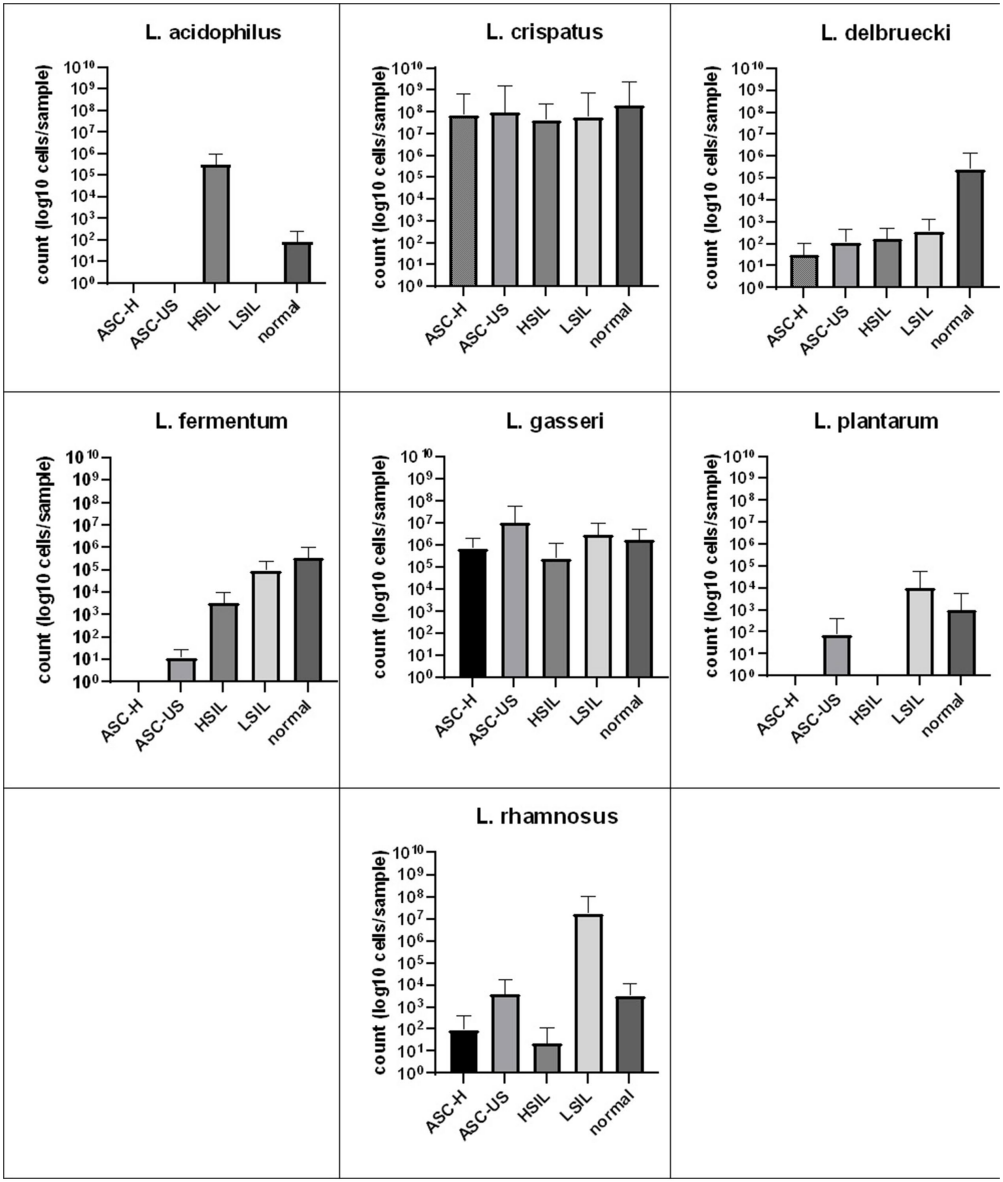
Comparison of the average number of LAB bacterial cells detected by real-time PCR from vaginal swabs in relation to cervical Pap smear result. SD, standard deviation; LSIL, low-grade squamous intraepithelial lesion; HSIL, high-grade squamous intraepithelial lesion; ASC-US, atypical squamous cells of undetermined significance; ASC-H, atypical squamous cells, cannot exclude a high-grade squamous intraepithelial lesion.

The quantitative real-time PCR analysis also demonstrated the numbers of *L. crispatus* and *L. gasseri* cells were the highest. Statistical analysis did not show any significant differences in the number of cells of each species between the studied subgroups of patients (Figure 2). What’s interesting, *L. acidophilus* was observed only in patients with HSIL and normal smear, while *L. plantarum* in women with ASC-US, LSIL and normal smear.

Moreover, analysis of the species distribution in different age groups showed that the abundance of *L. delbrueckii* significantly increased, whereas that of *L. gasseri* decreased, with age (Table 4). Statistical analysis of *L. gasseri* in three age groups also showed a statistically significantly higher abundance of this bacterium in swabs taken from patients aged 18–29 years (*p* = 0.0046). *L. delbrueckii* was significantly more abundant in swabs obtained from the oldest group of patients (47–72 years) (*p* = 0.057).

**Table 4:** Analysis of LAB species distribution according to participant age.

	Age groups, *n* (%)			*p* value
	18–29 (*n* = 26)	30–46 (*n* = 45)	47–72 (*n* = 29)	
*L. acidophilus*	0	2 (4.4)	0	0.29
*L. crispatus*	20 (76.9)	38 (84.4)	25 (86.2)	0.62
*L. delbrueckii*	2 (7.7)	12 (26.7)	11 (37.9)	0.06
*L. fermentum*	2 (7.7)	4 (8.9)	3 (10.3)	0.94
*L. gasseri*	26 (100)	43 (95.6)	24 (82.8)	0.005
*L. plantarum*	0	3 (6.7)	1 (3.45)	0.38
*L. rhamnosus*	1 (3.85)	8 (17.8)	6 (20.7)	0.17

*Lactobacillus acidophilus* (6.1%) and *L. fermentum* (18.2%) were found significantly more frequent among women living in rural areas than among those living in urban areas (*L. acidophilus* 0%, *L. fermentum* 4.5%) (Table 5). Also, the abundance of *L. acidophilus* cells (range 0–5.7×10^6^ vs. 0, *p* = 0.044) and *L. fermentum* cells (range 0–7.2×10^7^ vs. 0) was higher in women living in rural areas than in those living in urban areas (0–1.6×10^6^, *p* = 0.029).

**Table 5:** LAB species distribution according to participant place of residence.

	Number of patients (%)		*p* value
	Rural (*n* = 33)	Urban (*n* = 67)	
*L. acidophilus*	2 (6.1)	0	**0.04**
*L. crispatus*	30 (90.1)	53 (79.1)	0.14
*L. delbrueckii*	7 (21.2)	18 (26.9)	0.54
*L. fermentum*	6 (18.2)	3 (4.5)	**0.03**
*L. gasseri*	31 (93.4)	62 (92.5)	0.80
*L. plantarum*	3 (9.1)	1 (1.5)	0.068
*L. rhamnosus*	6 (18.2)	9 (13.4)	0.53

Bold values mean statistical significance.

There were no statistically significant differences in the vaginal microbiota in patients HPV positive (23 patients) and without HPV infection (23 patients), in those with and without vaginitis within the last 3 months, and between premenopausal and post-menopausal participants (Table 6).

**Table 6:** Occurrence of LAB species in patients by HPV, vaginitis, and menopausal status.

	HPV-positive	HPV-negative	*p* value	Vaginitis-positive	Vaginitis-negative	*p* value	Pre menopausal	Post menopausal	*p* value
	*N* = 23	*N* = 23		*N* = 28	*N* = 72		*N* = 82	*N* = 18	
*L. acidophilus*	0 (0)	0	1.0	1 (3.6)	1 (1.4)	0.48	2 (2.4)	0 (0)	1.0
*L. crispatus*	21 (91.3)	17 (73.9)	0.24	20 (71.4)	63 (87.5)	0.075	68 (82.9)	15 (83.3)	1.0
*L. delbrueckii*	5 (21.7)	3 (13.0)	0.70	8 (28.6)	17 (23.6)	0.61	18 (21.9)	7 (38.9)	0.14
*L. fermentum*	2 (8.7)	3 (13.0)	1.0	2 (7.1)	7 (9.7)	1.0	7 (8.5)	2 (11.1)	0.66
*L. gasseri*	22 (95.7)	20 (87.0)	0.61	26 (92.9)	67 (93.1)	1.0	77 (93.9)	16 (88.9)	0.61
*L. plantarum*	0 (0)	1 (4.4)	1.0	2 (7.1)	2 (2.8)	0.31	4 (4.9)	0 (0)	1.0
*L. rhamnosus*	3 (13.0)	4 (17.4)	1.0	4 (14.3)	11 (15.3)	1.0	11 (13.4)	4 (22.2)	0.46

### 3.3. Correlations between different *Lactobacilli* species

**Figure 3:**
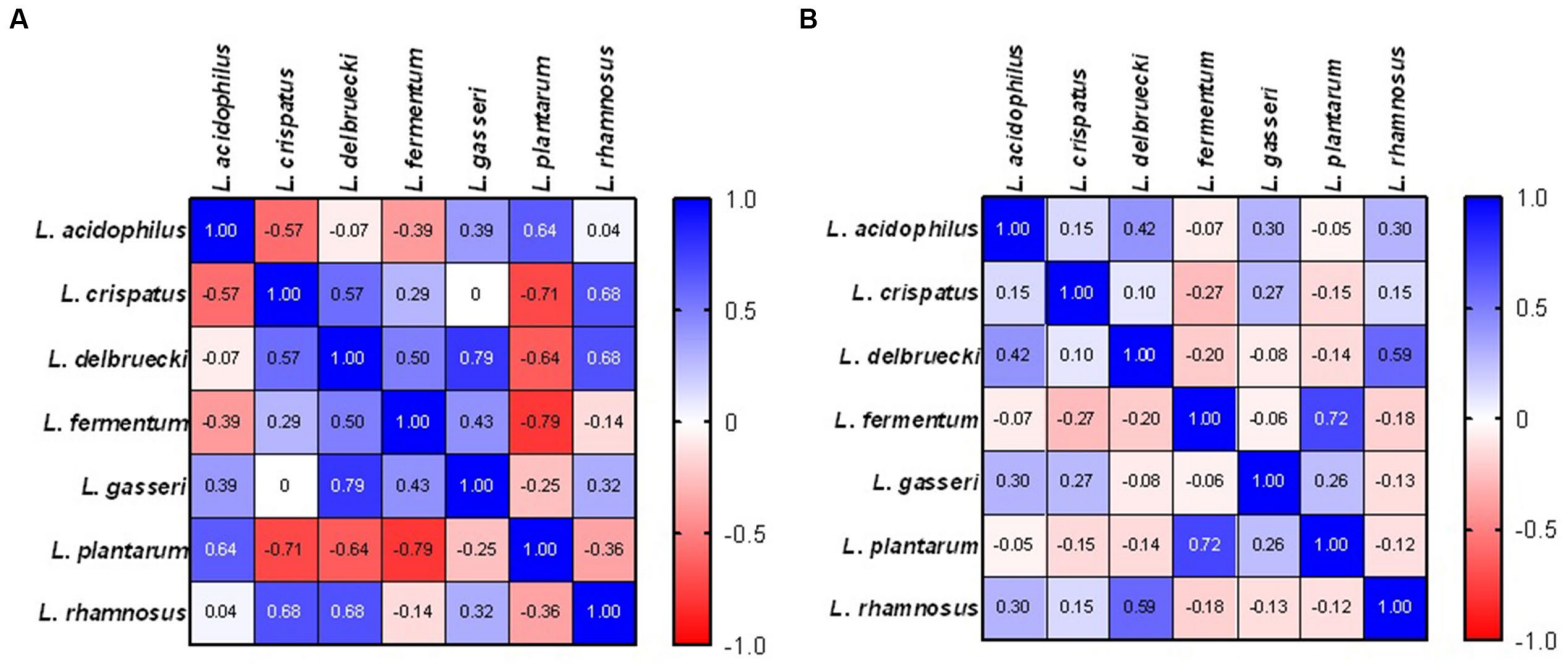
Correlations indicating the co-occurrence of individual LAB species in patients with abnormal cervical Pap smear result **(A)** and healthy control **(B)**.

In patients with abnormal cervical Pap smear result significant positive correlations were identified for *L. delbrueckii* and *L. gasseri* (*r* = 0.79, *p* = 0.048), and negative correlation between *L. fermentum* and *L. plantarum* (*r* = −0.79, *p* = 0.048). In healthy control group, significant correlation between *L. delbrueckii* and *L. rhamnosus* (*r* = 0.59, *p* = 0.005) and *L. pantarum* and *L. fermentum* (*r* = 0.72, *p* = 0.0002) was detected indicating the coexistence of these species (Figure 3).

## 4. Discussion

The vaginal microbiome plays a vital role in keeping the female reproductive system healthy. Bacterial vaginosis is a state in which the natural balance of bacteria in the vagina is disturbed, which has been linked to a wide variety of health problems, including a higher risk of sexually transmitted diseases, cystitis, postoperative complications, infertility, miscarriages, premature births, intrauterine infections, cervical and uterus infections, dysplasia, and cervical cancer. Also, cervical microbiota is crucial since the cervix is the place of infections caused by pathogens such as human immunodeficiency virus (HIV) and HPV, *Chlamydia trachomatis*, or *Neisseria gonorrhoeae*.

In our present study, vaginal microbiota of most participants in both groups was dominated by two of the seven LABs investigated. Three species were observed in 17% of the examined participants, four in 11%, and five in 3% of participants. This study revealed the presence of LAB belonging to seven species (*L. acidophilus*, *L. crispatus*, *L. delbrueckii*, *L. fermentum.*, *L. gasseri*, *L. plantarum*, and *L. rhamnosus*) in the material from vaginal swabs, with *L. gasseri* being most frequently detected, and *L. crispatus being most abundant one.* The dominance of *Lactobacillus crispatus* and *L. gasseri* is observed in community state types (CST) I and II group, respectively (Ravel et al., 2011). The presence of *L. gasseri* was suggested to be associated with rapid clearance of acute HPV infection (Brotman et al., 2014). It has to be noted that HPV infection itself was demonstrated to modify the mucosal metabolism and host immunity, thus promoting changes in the vaginal microbiota (Scott et al., 1999). The results of studies indicate that *Lactobacillus* species are capable of adhering tightly to the vaginal epithelium, covering its surface, thus protecting the vagina from colonization by pathogenic microorganisms (Petricevic et al., 2014; Mitra et al., 2016). The presence of microbiome dominated by *Lactobacilli* is related with well-balanced immune-tolerant vaginal microenvironment (Donnarumma et al., 2014). However, it was observed that not all the species equally contributed.

*Lactobacillus gasseri* was the most frequently detected species in our study, whereas *L. crispatus* was the most abundant one. Similar results were obtained by De Backer et al. (2007), who examined 71 vaginal swabs collected from Belgian women. They demonstrated that, among four *Lactobacillus* species (*L. crispatus*, *L. iners*, *L. jensenii*, and *L. gasseri*), *L. crispatus* was the most common (detected in 93% of the samples); *L. iners* was detected in 75% of swabs, *L. gasseri* in 73%, and *L. jensenii* in 46%. In another study, *L. crispatus* was the most abundant *Lactobacillus* species in the samples collected from healthy women and from women with bacterial vaginosis (Zozaya-Hinchliffe et al., 2010). In the vaginal and cervical swabs of 96 women from Florida, *L. iners* (55%), *L. crispatus* (29%), *L. gasseri* (13%), and *L. jensenii* (13%) were the most common species. In another American study, *L. iners* was the most abundant species in healthy women and in those with bacterial vaginosis (Pavlova et al., 2002). A meta-analysis by Norenhag et al. (2020) showed that *Lactobacillus* in the vaginal microbiome of 55% of HPV-positive and 38% of HPV-negative women was sparse. However, in 44% of HPV-positive and 58% of HPV-negative women, the vaginal microbiome was rich in *L. crispatus* and *L. iners*.

In this study, we failed to observe statistically significant differences between groups of patients with normal Pap smear results and those with ASC-US, ASC-H, LSIL, and HSIL. The number of cells of a given bacteria appears to be of higher importance than the presence itself. In our study, quantitative analysis demonstrated the highest abundance of *L. crispatus* and *L. gasseri* cells. However, again we failed to show any significant differences in cells number of each species between the studied subgroups of patients. No statistically significant differences in the vaginal microbiota in HPV positive and HPV negative patients, in those with and without vaginitis within the last 3 months, and between premenopausal and post-menopausal participants were observed. In contrast, a study among African women showed that a cervical microbiota dominated by *Lactobacillus* spp. (*L. crispatus, L. inners*) was associated with a lower incidence of HIV, herpes simplex virus 2 (HSV-2), and high-risk HPV strains (Borgdorff et al., 2014). The abundance of *L. gasseri* and *L. iners* in the vagina has been associated with the rapid resolution of HPV infection. By contrast, low levels of *Lactobacillus* spp. and high levels of *Atopobium* spp. have been associated with slower resolution of HPV infection (Gao et al., 2013; Lee et al., 2013; Brotman et al., 2014). Vaginal infections with *C. trachomatis* have also been suggested to enhance women’s susceptibility to HPV infections via altering the composition of the vaginal microbiota (Silva et al., 2014). Several studies have focused on the correlation between the vaginal microbiome and cervical dysplasia. Overall, a vaginal microbiome rich in *L. crispatus* occurs in a healthy state (Oh et al., 2015; Audirac-Chalifour et al., 2016; Seo et al., 2016), whereas *L. iners* seems to be related to cervical cancer either alone or associated with HPV infection (Oh et al., 2015; Piyathilake et al., 2016; Seo et al., 2016). However, another study showed the opposite relationship, with *L. iners* associated with a reduced risk of cervical intraepithelial neoplasia and cervical cancer (Audirac-Chalifour et al., 2016). Other *Lactobacillus* species have also shown differing associations with cervical dysplasia (Mitra et al., 2015; Piyathilake et al., 2016).

In this study, we observed a strong positive correlation between *L. delbrueckii* and *L. gasseri* as well as negative correlation between *L. fermentum* and *L. plantarum* in patients with abnormal cervical Pap smear. In turn, in healthy control group, the presence of *L. delbrueckii* correlated with *L. rhamnosus* as well as between *L. pantarum* and *L. fermentum.* We did not observe correlation between *L. crispatus* and any other species. Similary, Gajer et al. (2012) reported that this bacteria is rarely coexisting with other bacterial species since it is either strongly dominant, or absent. Moreover, it was suggested that *L. crispatus* is associated with protection from pathogens and is the least likely to transition into CST IV. Women with such microbiota are less likely to become infected with Herpes simplex virus (HSV)-2 and HIV, as well HPV (Borgdorff et al., 2014). Vaginal microbial communities exist in a state of dynamic equilibrium which provides resilience (Ravel et al., 2011). The coexistence of various *Lactobacillus* species in the vaginal milieu enables the maintenance of homeostatic stage. Bacteria show different pH preference, growth patterns and lactic acid production capability (Mehta et al., 2020). The coexistence preferences have been shown to play a key role in the modulation of bacterial taxa composition in the vaginal ecosystem (Verstraelen et al., 2009).

In our study, *L. acidophilus* was observed only in patients with HSIL and normal smear, while *L. plantarum* in women with ASC-US, LSIL and normal smear. Similarly, Kwasniewski et al. (2018) observed the prevalence of *Lactobacillus acidophilus,* but also *Lactobacillus iners* and *Lactobacillus crispatus* in women diagnosed with HSIL. Moreover, in their study *Lactobacillus acidophilus* and *Lactobacillus iners* were predominant in CST diagnosed with LSIL HPV(+), while *Lactobacillus crispatus* was absent in smears.

Moreover, in our study, the analysis of the species distribution in different age groups showed that the abundance of *L. delbrueckii* significantly increased, whereas that of *L. gasseri* decreased, with age. *L. acidophilus* and *L. fermentum* were significantly more prevalent and the number of their cells were much higher among women living in rural areas compared with those living in urban areas.

Apart from the area of residence, the composition of the vaginal microbiota differs between various races. The composition of vaginal microbiome differs between women in different geographic regions therefore, the comparison of results obtained in studies of various ethnic groups show discrepancies. Studies have shown that the variety of *Lactobacillus* species in the vagina is limited to three to seven species and strictly depends on the population of the studied women (Pavlova et al., 2002; Jin et al., 2007). Studies of Polish women indicated that their vaginal regions are most frequently colonized by three species of *Lactobacillus* (Pytka et al., 2019), with *L. crispatus*, *L. gasseri*, and *L. jensenii* being the most frequent. However, vaginal microbiota of Swedish healthy women is dominated in 78% by single LAB species. The most prevalent are: *L. crispatus, L. gasseri, L. iners* and *L. jensenii* (Vasquez et al., 2002). All isolated vaginal *Lactobacillus* strains identified in German women belonged to three species: *Lactobacillus crispatus* (56%), *Lactobacillus jensenii* (26%), and *Lactobacillus gasseri* (18%) (Hütt et al., 2016). Researchers from Turkey obtained similar results. They reported that, among 56 identified species 32% belonged to *L. crispatus*, 30% to *L. gasseri*, and 13.8% to *L. jensenii* (Eryilmaz et al., 2018). In an Italian study, 261 vaginal *Lactobacillus* species were detected, with *L. gasseri* (28%), *L. salivarius* (20%), *L. crispatus* (18%), *L. helveticus* (13%), *L. fermentum* (10%), and *L. rhamnosus* (10%) being the most common (Pino et al., 2019). The prevalence of *L. crispatus* and *L. iners* in vaginal was found to be lower in German females (42.4%), Indonesian population (45.0%), Kenyan women (34.4%) and Afro-Caribbean women (26.1%) compared to African American women (91.8%) (Roachford et al., 2022). Similarly, Ravel et al. (2011) observed that vaginal bacterial communities in North American women representing four ethnic groups (white, black, Hispanic, and Asian) were dominated by species of *Lactobacillus*. *Lactobacillus* groups I, II, III, and V were identified in 80.2 and 89.7% of Asian and white women, respectively, however, in only 59.6 and 61.9% of Hispanic and black women, respectively. Moreover, it was revealed that community group IV (diverse group) was overrepresented in Hispanic (34.3%) and black (38.9%) women in comparison to Asian (17.6%) and white (9.3%) women which suggest that such vaginal bacterial communities appear normal in black and Hispanic women (Ravel et al., 2011). The higher prevalence of communities not dominated by *Lactobacillus* sp. (cluster IV) in Hispanic and black females could be associated with differences in vaginal pH (5.0 ± 0.59 in Hispanic) and (pH 4.7 ± 1.04 in black females) in comparison to Asian (pH 4.4 ± 0.59) and white (pH 4.2 ± 0.3) women. Therefore, it appears that vaginal microbiome can change under factors such as glycogen content in epithelial cells, pH, hormone levels, damage caused by sexual intercourse, type of contraception, age, and antibiotic therapy (Valenta, 2005). As commonly known, the v environment is acidic at a pH of 4–4.5. 93 species of the genus *Lactobacillus* producing lactic acid are responsible for creating such conditions, which hinder colonization by pathogens. Nevertheless, after menopause, when estrogen levels decrease, the vaginal microbiome changes, species diversity increases, and *Lactobacillus* spp. co-exist with other species. As a result, vaginal pH and susceptibility to vaginal infections increase (Miller et al., 2016).

This study also shows that HPV infection is not recognized by many females as important risk factor for cervical cancer. Only less than 50% of women in this study have ever been tested for HPV, and none of them have been vaccinated against it. Persistent oncogenic HPV infections are essential for cervical oncogenesis. Studies indicate the involvement of the vaginal microbiota in the persistence or resolution of HPV infection. In a study on twins, Lee et al. (2013) showed that, when one of the twins is HPV-positive, there is a significant change in the vaginal microbiota, an increase in species diversity, and a decrease in the number of *Lactobacillus* spp. bacterial cells, compared with the healthy twin. Mitra et al. (2016) showed that the increasing diversity of the vaginal microbiome and the decreasing number of *Lactobacillus* cells are associated with the progression of cervical pathological changes.

The study was limited by the random selection of the study population and the limited possibility of extrapolating the results to the general population. We also did not include patients after HPV vaccination, which is not a very popular vaccination in women over 18 years of age in Poland. Further research is needed, particularly to determine the composition of the vaginal microbiota in women who have been vaccinated against HPV. Next limitation could be the use the assay based on qPCR amplification of a species-specific genetic region of the relevant microbe. Then, some species reported in other studies carried out with the use of next generation sequencing technique were not detected, i.e., *L. iners* or *L. jensenii*. Although the study has limitations, the results have the potential to be implemented in practice. There are increasing numbers of reports that vaginal application of certain dietary supplements can affect the vaginal microbiome in patients with abnormal cytology, resulting in regression of abnormal cytology and a return to normal cytology. This could be related to the influence of the vaginal microbiome bacteria on HPV or due to another mechanism. Determining the exact composition of the vaginal microbiome of healthy women in Poland might influence the use of this knowledge in the production of these preparations.

## 5. Conclusion

The data obtained in this study suggest that two *Lactobacillus* species (*L. gasseri* and *L. crispatus*) were predominant in the vaginal microbiota of the studied population of Polish women, regardless of smear test results. In this study, patient age and place of residence were associated with the diversity of *Lactobacillus* spp. in the vaginal microbiota. The abundance of *L. delbrueckii* in the vaginal microbiota increased, whereas that of *L. gasseri* decreased, with age. *L. acidophilus* and *L. fermentum* were significantly more frequently detected in women living in rural versus urban areas. The degree of dysplastic changes in the endothelium or the presence of a group of atypical cervical stratified epithelial cells was not associated with significant changes in the studied vaginal bacteria. A similar lack of significant differences in the qualitative and quantitative composition of the vaginal microbiota was also observed when analyzing associations with menopausal status, HPV infection, or vaginitis within 3 months before the study.

## Data availability statement

The data analyzed in this study is subject to the following licenses/restrictions: Available upon request. Requests to access these datasets should be directed to bbarczynski@poczta.onet.pl.

## Ethics statement

The studies involving humans were approved by Bioethics Committee of Medical University in Lublin KE – 0254/174/2014. The studies were conducted in accordance with the local legislation and institutional requirements. The participants provided their written informed consent to participate in this study.

## Author contributions

KF: Conceptualization, Investigation, Visualization, Writing – original draft, Writing – review & editing. BB: Conceptualization, Investigation, Visualization, Writing – original draft, Writing – review & editing, Data curation, Formal analysis, Project administration. RS: Data curation, Formal analysis, Investigation, Methodology, Software, Writing – review & editing. AK: Writing – review & editing. AM: Conceptualization, Formal analysis, Funding acquisition, Investigation, Methodology, Project administration, Resources, Software, Supervision, Validation, Visualization, Writing – review & editing. JK: Conceptualization, Formal analysis, Funding acquisition, Investigation, Resources, Supervision, Validation, Visualization, Writing – review & editing. EW: Writing – review & editing. IK-G: Conceptualization, Data curation, Formal analysis, Funding acquisition, Investigation, Investigation, Project administration, Resources, Software, Supervision, Validation, Visualization, Writing – original draft, Writing – review & editing.

## References

[ref1] Arbyn M. Weiderpass E. Bruni L. de Sanjosé S. Saraiya M. Ferlay J.. (2020). Estimates of incidence and mortality of cervical cancer in 2018: a worldwide analysis. Lancet Glob. Health 8, e191–e203. doi: 10.1016/S2214-109X(19)30482-6, PMID: 31812369 PMC7025157

[ref2] Audirac-Chalifour A. Torres-Poveda K. Bahena-Román M. Téllez-Sosa J. Martínez-Barnetche J. Cortina-Ceballos B.. (2016). Cervical microbiome and cytokine profile at various stages of cervical cancer: a pilot study. PLoS One 11:e0153274. doi: 10.1371/journal.pone.0153274, PMID: 27115350 PMC4846060

[ref3] Borgdorff H. Tsivtsivadze E. Verhelst R. Marzorati M. Jurriaans S. Ndayisaba G. F.. (2014). *Lactobacillus*-dominated cervicovaginal microbiota associated with reduced HIV/STI prevalence and genital HIV viral load in African women. ISME J. 8, 1781–1793. doi: 10.1038/ismej.2014.26, PMID: 24599071 PMC4139719

[ref4] Borges S. Silva J. Teixeira P. (2014). The role of *Lactobacilli* and probiotics in maintaining vaginal health. Arch. Gynecol. Obstet. 289, 479–489. doi: 10.1007/s00404-013-3064-9 24170161

[ref5] Brotman R. M. Shardell M. D. Gajer P. Tracy J. K. Zenilman J. M. Ravel J.. (2014). The interplay between the temporal dynamics of the vaginal microbiota and human papillomavirus detection. J. Infect. Dis. 210, 1723–1733. doi: 10.1093/infdis/jiu330, PMID: 24943724 PMC4296189

[ref6] Byun R. Nadkarni M. A. Chhour K. L. Martin F. E. Jacques N. A. Hunter N. (2004). Quantitative analysis of diverse *Lactobacillus* species present in advanced dental caries. J. Clin. Microbiol. 42, 3128–3136. doi: 10.1128/JCM.42.7.3128-3136.2004, PMID: 15243071 PMC446321

[ref7] Champer M. Wong A. M. Champer J. Brito I. L. Messer P. W. Hou J. Y.. (2018). The role of the vaginal microbiome in gynaecological cancer. BJOG 125, 309–315. doi: 10.1111/1471-0528.14631, PMID: 28278350

[ref8] Chatterjee T. Gill S. S. Rac R. (2000). Standardization of cervical/vaginal cytopathology reporting: the Bethesda system (TBS) for reporting cervical/vaginal cytologic diagnoses. Med. J. Armed Forces India 56, 45–49. doi: 10.1016/S0377-1237(17)30090-4, PMID: 28790644 PMC5531959

[ref9] De Backer E. Verhelst R. Verstraelen H. Alqumber M. A. Burton J. P. Tagg J. R.. (2007). Quantitative determination by real-time PCR of four vaginal *Lactobacillus* species, Gardnerella vaginalis and *Atopobium vaginae* indicates an inverse relationship between *L. gasseri* and *L. iners*. BMC Microbiol. 7, 1–13. doi: 10.1186/1471-2180-7-115, PMID: 18093311 PMC2233628

[ref10] Dominguez-Bello M. G. Godoy-Vitorino F. Knight R. Blaser M. J. (2019). Role of the microbiome in human development. Gut 68, 1108–1114. doi: 10.1136/gutjnl-2018-317503, PMID: 30670574 PMC6580755

[ref11] Donnarumma G. Molinaro A. Cimini D. De Castro C. Valli V. De Gregorio V.. (2014). *Lactobacillus crispatus* L1: high cell density cultivation and exopolysaccharide structure characterization to highlight potentially beneficial effects against vaginal pathogens. BMC Microbiol. 14:137. doi: 10.1186/1471-2180-14-137, PMID: 24884965 PMC4054921

[ref12] Eryilmaz M. Gurpinar S. S. Palabiyik I. M. Guriz H. Gerceker D. (2018). Molecular identification and antimicrobial activity of vaginal *Lactobacillus* sp. Curr. Pharm. Biotechnol. 19, 1241–1247. doi: 10.2174/1389201020666190110164123, PMID: 30636598

[ref13] Flores G. E. Caporaso J. G. Henley J. B. Rideout J. R. Domogala D. Chase J.. (2014). Temporal variability is a personalized feature of the human microbiome. Genome Biol. 15, 1–13. doi: 10.1186/s13059-014-0531-y PMC4252997 25517225

[ref14] Gajer P. Brotman R. M. Bai G. Sakamoto J. Schütte U. M. E. Zhong X.. (2012). Temporal dynamics of the human vaginal microbiota. Sci. Transl. Med. 4, 132ra52–32ra52. doi: 10.1126/scitranslmed.3003605 PMC3722878 22553250

[ref15] Gao W. Weng J. Gao Y. Chen X. (2013). Comparison of the vaginal microbiota diversity of women with and without human papillomavirus infection: a cross-sectional study. BMC Infect. Dis. 13, 1–10. doi: 10.1186/1471-2334-13-271, PMID: 23758857 PMC3684509

[ref16] Gillet E. Meys J. F. Verstraelen H. Bosire C. De Sutter P. Temmerman M.. (2011). Bacterial vaginosis is associated with uterine cervical human papillomavirus infection: a meta-analysis. BMC Infect. Dis. 11, 1–9. doi: 10.1186/1471-2334-11-10, PMID: 21223574 PMC3023697

[ref17] Gillet E. Meys J. F. Verstraelen H. Verhelst R. De Sutter P. Temmerman M.. (2012). Association between bacterial vaginosis and cervical intraepithelial neoplasia: systematic review and meta-analysis. PLoS One 7:e45201. doi: 10.1371/journal.pone.0045201, PMID: 23056195 PMC3462776

[ref18] Guo Y. L. You K. Qiao J. Zhao Y. M. Geng L. (2012). Bacterial vaginosis is conducive to the persistence of HPV infection. Int. J. STD AIDS 23, 581–584. doi: 10.1258/ijsa.2012.011342, PMID: 22930296

[ref19] Haarman M. Knol J. (2006). Quantitative real-time PCR analysis of fecal *Lactobacillus* species in infants receiving a prebiotic infant formula. Appl. Environ. Microbiol. 72, 2359–2365. doi: 10.1128/AEM.72.4.2359-2365.2006, PMID: 16597930 PMC1448991

[ref20] Hütt P. Lapp E. Štšepetova J. Smidt I. Taelma H. Borovkova N.. (2016). Characterisation of probiotic properties in human vaginal *Lactobacilli* strains. Microb. Ecol. Health Dis. 27:30484. doi: 10.3402/mehd.v27.30484, PMID: 27527701 PMC4985617

[ref21] Jin L. Tao L. Pavlova S. I. So J. S. Kiwanuka N. Namukwaya Z.. (2007). Species diversity and relative abundance of vaginal lactic acid bacteria from women in Uganda and Korea. J. Appl. Microbiol. 0:3147. doi: 10.1111/j.1365-2672.2006.03147.x, PMID: 17381754

[ref22] King C. C. Jamieson D. J. Wiener J. Cu-Uvin S. Klein R. S. Rompalo A. M.. (2011). Bacterial vaginosis and the natural history of human papillomavirus. Infect. Dis. Obstet. Gynecol. 2011, 1–8. doi: 10.1155/2011/319460, PMID: 21869857 PMC3159014

[ref23] Kwasniewski W. Wolun-Cholewa M. Kotarski J. Warchol W. Kuzma D. Kwasniewska A.. (2018). Microbiota dysbiosis is associated with HPV-induced cervical carcinogenesis. Oncol. Lett. 16, 7035–7047. doi: 10.3892/ol.2018.9509, PMID: 30546437 PMC6256731

[ref24] Lee J. E. Lee S. Lee H. Song Y. M. Lee K. Han M. J.. (2013). Association of the vaginal microbiota with human papillomavirus infection in a Korean twin cohort. PLoS One 8:e63514. doi: 10.1371/journal.pone.0063514, PMID: 23717441 PMC3661536

[ref25] Liu M. B. Xu S. R. He Y. Deng G. H. Sheng H. F. Huang X. M.. (2013). Diverse vaginal microbiomes in reproductive-age women with vulvovaginal candidiasis. PLoS One 8:e79812. doi: 10.1371/journal.pone.0079812 24265786 PMC3827160

[ref26] MacIntyre D. A. Chandiramani M. Lee Y. S. Kindinger L. Smith A. Angelopoulos N.. (2015). The vaginal microbiome during pregnancy and the postpartum period in a European population. Sci. Rep. 5, 1–9. doi: 10.1038/srep08988, PMID: 25758319 PMC4355684

[ref27] Marchesi J. R. Ravel J. (2015). The vocabulary of microbiome research: a proposal. Microbiome 3, 1–3. doi: 10.1186/s40168-015-0094-5 26229597 PMC4520061

[ref28] Mehta O. Ghosh T. S. Akansha Kothidar M. Gowtham R. Mitra R. Kshetrapal P.. (2020). Vaginal microbiome of pregnant Indian women: insights into the genome of dominant *Lactobacillus* species. Microb. Ecol. 80, 487–499. doi: 10.1007/s00248-020-01501-0 32206831

[ref29] Miller E. A. Beasley D. E. Dunn R. R. Archie E. A. (2016). *Lactobacilli* dominance and vaginal pH: why is the human vaginal microbiome unique? Front. Microbiol. 7, 1–13. doi: 10.3389/fmicb.2016.01936, PMID: 28008325 PMC5143676

[ref30] Mitra A. MacIntyre D. A. Lee Y. S. Smith A. Marchesi J. R. Lehne B.. (2015). Cervical intraepithelial neoplasia disease progression is associated with increased vaginal microbiome diversity. Sci. Rep. 5, 1–11. doi: 10.1038/srep16865, PMID: 26574055 PMC4648063

[ref31] Mitra A. MacIntyre D. A. Marchesi J. R. Lee Y. S. Bennett P. R. Kyrgiou M. (2016). The vaginal microbiota, human papillomavirus infection and cervical intraepithelial neoplasia: what do we know and where are we going next? Microbiome 4, 58–15. doi: 10.1186/s40168-016-0203-0, PMID: 27802830 PMC5088670

[ref32] Moreno I. Simon C. (2018). Deciphering the effect of reproductive tract microbiota on human reproduction. Reprod. Med. Biol. 18, 40–50. doi: 10.1002/rmb2.12249 30655720 PMC6332752

[ref34] Norenhag J. Du J. Olovsson M. Verstraelen H. Engstrand L. Brusselaers N. (2020). The vaginal microbiota, human papillomavirus and cervical dysplasia: a systematic review and network meta-analysis. BJOG 127, 171–180. doi: 10.1111/1471-0528.15854 31237400

[ref35] Oh H. Y. Kim B. S. Seo S. S. Kong J. S. Lee J. K. Park S. Y.. (2015). The association of uterine cervical microbiota with an increased risk for cervical intraepithelial neoplasia in Korea. Clin. Microbiol. Infect. 21, 674.e1–674.e9. doi: 10.1016/j.cmi.2015.02.026, PMID: 25752224

[ref36] Pavlova S. I. Kilic A. O. Kilic S. S. So J. S. Nader-Macias M. E. Simoes J. A.. (2002). Genetic diversity of vaginal *Lactobacilli* from women in different countries based on 16S rRNA gene sequences. J. Appl. Microbiol. 92, 451–459. doi: 10.1046/j.1365-2672.2002.01547.x, PMID: 11872120

[ref37] Petricevic L. Domig K. J. Nierscher F. J. Sandhofer M. J. Fidesser M. Krondorfer I.. (2014). Characterisation of the vaginal *Lactobacillus* microbiota associated with preterm delivery. Sci. Rep. 4:5136. doi: 10.1038/srep05136, PMID: 24875844 PMC4038809

[ref38] Pino A. Bartolo E. Caggia C. Cianci A. Randazzo C. L. (2019). Detection of vaginal *Lactobacilli* as probiotic candidates. Sci. Rep. 9, 3355–3310. doi: 10.1038/s41598-019-40304-3, PMID: 30833631 PMC6399336

[ref39] Piyathilake C. J. Ollberding N. J. Kumar R. Macaluso M. Alvarez R. D. Morrow C. D. (2016). Cervical microbiota associated with higher grade cervical intraepithelial neoplasia in women infected with high-risk human papillomaviruses. Cancer Prev. Res. 9, 357–366. doi: 10.1158/1940-6207.CAPR-15-0350, PMID: 26935422 PMC4869983

[ref40] Pytka M. Kordowska-Wiater M. Jarocki P. (2019). Microbiome of the women’s genital system. Adv. Microbiol. 58, 227–236. doi: 10.21307/PM-2019.58.3.227

[ref41] Ravel J. Gajer P. Abdo Z. Schneider G. M. Koenig S. S. McCulle S. L.. (2011). Vaginal microbiome of reproductive-age women. Proc. Natl. Acad. Sci. U. S. A. 108, 4680–4687. doi: 10.1073/pnas.1002611107, PMID: 20534435 PMC3063603

[ref42] Requena T. Velasco M. (2021). The human microbiome in sickness and in health. Rev. Clin. Esp. 221, 233–240. doi: 10.1016/j.rceng.2019.07.018, PMID: 33998505

[ref43] Roachford O. S. E. Alleyne A. T. Nelson K. E. (2022). Insights into the vaginal microbiome in a diverse group of women of African, Asian and European ancestries. PeerJ 10:e14449. doi: 10.7717/peerj.14449, PMID: 36518275 PMC9744153

[ref44] Scott M. Stites D. P. Moscicki A. B. (1999). Th1 cytokine patterns in cervical human papillomavirus infection. Clin. Diagn. Lab. Immunol. 6, 751–755. doi: 10.1128/CDLI.6.5.751-755.1999 10473530 PMC95767

[ref45] Sender R. Fuchs S. Milo R. (2016). Are we really vastly outnumbered? Revisiting the ratio of bacterial to host cells in humans. Cells 164, 337–340. doi: 10.1016/j.cell.2016.01.013, PMID: 26824647

[ref46] Seo S. S. Oh H. Y. Lee J. K. Kong J. S. Lee D. O. Kim M. K. (2016). The combined effect of diet and cervical microbiome on the risk of cervical intraepithelial neoplasia. Clin. Nutr. 35, 1434–1441. doi: 10.1016/j.clnu.2016.03.019, PMID: 27075319

[ref47] Silva J. Cerqueira F. Medeiros R. (2014). *Chlamydia trachomatis* infection: implications for HPV status and cervical cancer. Arch. Gynecol. Obstet. 289, 715–723. doi: 10.1007/s00404-013-3122-3, PMID: 24346121

[ref48] Tachedjian G. Aldunate M. Bradshaw C. S. Cone R. A. (2017). The role of lactic acid production by probiotic *Lactobacillus* species in vaginal health. Res. Microbiol. 168, 782–792. doi: 10.1016/j.resmic.2017.04.001, PMID: 28435139

[ref49] Turnbaugh P. J. Ley R. E. Hamady M. Fraser-Liggett C. M. Knight R. Gordon J. I. (2007). The human microbiome project. Nature 449, 804–810. doi: 10.1038/nature06244, PMID: 17943116 PMC3709439

[ref50] Valenta C. (2005). The use of mucoadhesive polymers in vaginal delivery. Adv. Drug Deliv. Rev. 57, 1692–1712. doi: 10.1016/j.addr.2005.07.004 16182407

[ref51] Vasquez A. Jakobsson T. Ahrne S. Forsum U. Molin G. (2002). Vaginal *Lactobacillus* flora of healthy Swedish women. J. Clin. Microbiol. 40, 2746–2749. doi: 10.1128/JCM.40.8.2746-2749.2002, PMID: 12149323 PMC120688

[ref52] Verstraelen H. Verhelst R. Claeys G. De Backer E. Temmerman M. Vaneechoutte M. (2009). Longitudinal analysis of the vaginal microflora in pregnancy suggests that *L. crispatus* promotes the stability of the normal vaginal microflora and that *L. gasseri* and/or *L. iners* are more conducive to the occurrence of abnormal vaginal microflora. BMC Microbiol. 9:116. doi: 10.1186/1471-2180-9-116, PMID: 19490622 PMC2698831

[ref53] Wang H. Jiang Y. Liang Y. Wei L. Zhang W. Li L. (2022). Observation of the cervical microbiome in the progression of cervical intraepithelial neoplasia. BMC Cancer 22:362. doi: 10.1186/s12885-022-09452-0, PMID: 35379200 PMC8981842

[ref9001] World Medical Association. (2013). Ethical principles for medical research involving human subjects. JAMA. 310, 2191–2194. doi: 10.1001/jama.2013.281053 24141714

[ref54] Wu S. Ding X. Kong Y. Acharya S. Huaqian W. Huang C.. (2021). The feature of cervical microbiota associated with the progression of cervical cancer among reproductive females. Gynecol. Oncol. 163, 348–357. doi: 10.1016/j.ygyno.2021.08.016, PMID: 34503848

[ref55] Zhang R. Daroczy K. Xiao B. Yu L. Chen R. Liao Q. (2012). Qualitative and semiquantitative analysis of *Lactobacillus* species in the vaginas of healthy fertile and postmenopausal Chinese women. J. Med. Microbiol. 61, 729–739. doi: 10.1099/jmm.0.038687-0 22301614

[ref56] Zozaya-Hinchliffe M. Lillis R. Martin D. H. Ferris M. J. (2010). Quantitative PCR assessments of bacterial species in women with and without bacterial vaginosis. J. Clin. Microbiol. 48, 1812–1819. doi: 10.1128/JCM.00851-09, PMID: 20305015 PMC2863870

